# Cushing’s Syndrome With Nocardiosis: A Case Report and a Systematic Review of the Literature

**DOI:** 10.3389/fendo.2021.640998

**Published:** 2021-03-29

**Authors:** Da Zhang, Yan Jiang, Lin Lu, Zhaolin Lu, Weibo Xia, Xiaoping Xing, Hongwei Fan

**Affiliations:** ^1^ Department of Endocrinology, Key Laboratory of Endocrinology of National Health Commission, Peking Union Medical College Hospital, Peking Union Medical College, Chinese Academy of Medical Science, Beijing, China; ^2^ Department of Endocrinology, Air Force Medical Center, Beijing, China; ^3^ Department of Infectious Diseases, Peking Union Medical College Hospital, Peking Union Medical College, Chinese Academy of Medical Science, Beijing, China

**Keywords:** Cushing’s syndrome, nocardiosis, infection, ectopic ACTH syndrome, Cushing’s disease

## Abstract

**Objective:**

To analyze and summarize the clinical characteristics, treatments, and prognosis of Cushing’s syndrome (CS) with nocardiosis.

**Methods:**

A patient in our hospital and additional 17 patients of CS with nocardiosis in the English literature were included in this study. Clinical characteristics, laboratory data, imaging studies, treatments, and prognosis were evaluated.

**Results:**

A 41-year-old man with CS was diagnosed and treated in our hospital. He had co-infections of nocardiosis and aspergillosis. Together with 17 patients of CS with nocardiosis in the English literature, 2 patients (11.1%) were diagnosed as Cushing’s disease (CD) while 16 (88.9%) were diagnosed or suspected as ectopic ACTH syndrome (EAS). The average 24hrUFC was 7,587.1 ± 2,772.0 μg/d. The average serum total cortisol and ACTH (8 AM) was 80.2 ± 18.7 μg/dl and 441.8 ± 131.8 pg/ml, respectively. The most common pulmonary radiologic findings in CT scan were cavitary lesions (10/18) and nodules (8/18). Co-infections were found in 33.3% (6/18) patients. The CS patients with co-infections had higher levels of ACTH (671.5 ± 398.2 *vs* 245.5 ± 217.1 pg/ml, *P* = 0.047), and 38.9% (7/18) patients survived through the antibiotic therapy and the treatment of CS. Patients with lower level of ACTH (survival *vs* mortality: 213.1 ± 159.0 *vs* 554.7 ± 401.0 pg/ml, P = 0.04), no co-infection, underwent CS surgery, and received antibiotic therapy for more than 6 months, had more possibilities to survive.

**Conclusions:**

Nocardia infection should be cautioned when a patient of CS presented with abnormal chest radiographs. The mortality risk factors for CS with nocardiosis are high level of ACTH and co-infections. We should endeavor to make early etiological diagnosis, apply long-term sensitive antibiotics and aggressive treatments of CS.

## Introduction

Endogenous Cushing’s syndrome (CS) is characterized by excessive elevation of glucocorticoid concentrations produced by adrenal cortex. It is generally divided into adrenocorticotropic hormone (ACTH)-dependent and ACTH-independent CS. The most common cause of CS is corticotropin-secreting pituitary adenoma that leads to Cushing’s disease (CD). The ectopic ACTH syndrome (EAS) accounts for 10 to 20% of ACTH-dependent CS (1).

Nocardiosis most frequently presents with pulmonary disease, followed by disseminated disease, extra-pulmonary disease [such as in the central nervous system and primary skin and soft tissue disease (2)]. Nocardiosis is regarded as an opportunistic infection, with the majority of infections occurring in immunocompromised patients, including those with long-term corticosteroid exposure, malignancy, human immunodeficiency virus (HIV) infection, and history of transplantation (3–7), associated with high mortality of 34.5–40% (3, 4). Xu L. et al. (8) reviewed 12 patients of nocardiosis in EAS patients. However, reports of nocardiosis in patients with other forms of CS were not included.

In this study, we presented a patient of nocardiosis with suspected EAS in our hospital and analyzed 17 patients of nocardiosis in CS reported in the literature to summarize the clinical characteristics, treatments, and prognosis of CS with nocardiosis.

## Methods

### Medical Information of This Case

We collected the clinical characteristics, laboratory data, imagings, and microbiology results of a patient of CS with nocardiosis in Peking Union Medical College Hospital (PUMCH). This study was approved by the Ethics Committee of PUMCH.

### Literature Review

A systematic literature review was conducted through searched PubMed, Web of Science, and Embase, finding all relevant and available articles published in English. MeSH terms included “Cushing’s syndrome,” “Nocardia Infections,” or “nocardiosis.” Original research, case reports, case series, or review articles published until October, 2020 with detail medical history and laboratory data were included. Studies which analyzed cases of exogenous CS were excluded.

### Statistical Analysis

Data management and analysis were performed using SPSS 20.0 (SPSS Inc., Chicago, IL, USA). Data were presented as proportions for categorical variables and mean SD or median (interquartile range) for continuous variables. Significant differences between groups for continuous variables were tested using a *t*-test or the nonparametric Mann–Whitney *U* test, as appropriate. *χ^2^* tests were used for comparisons of categorical data.

## Results

### Case Presentation

**Figure 1:**
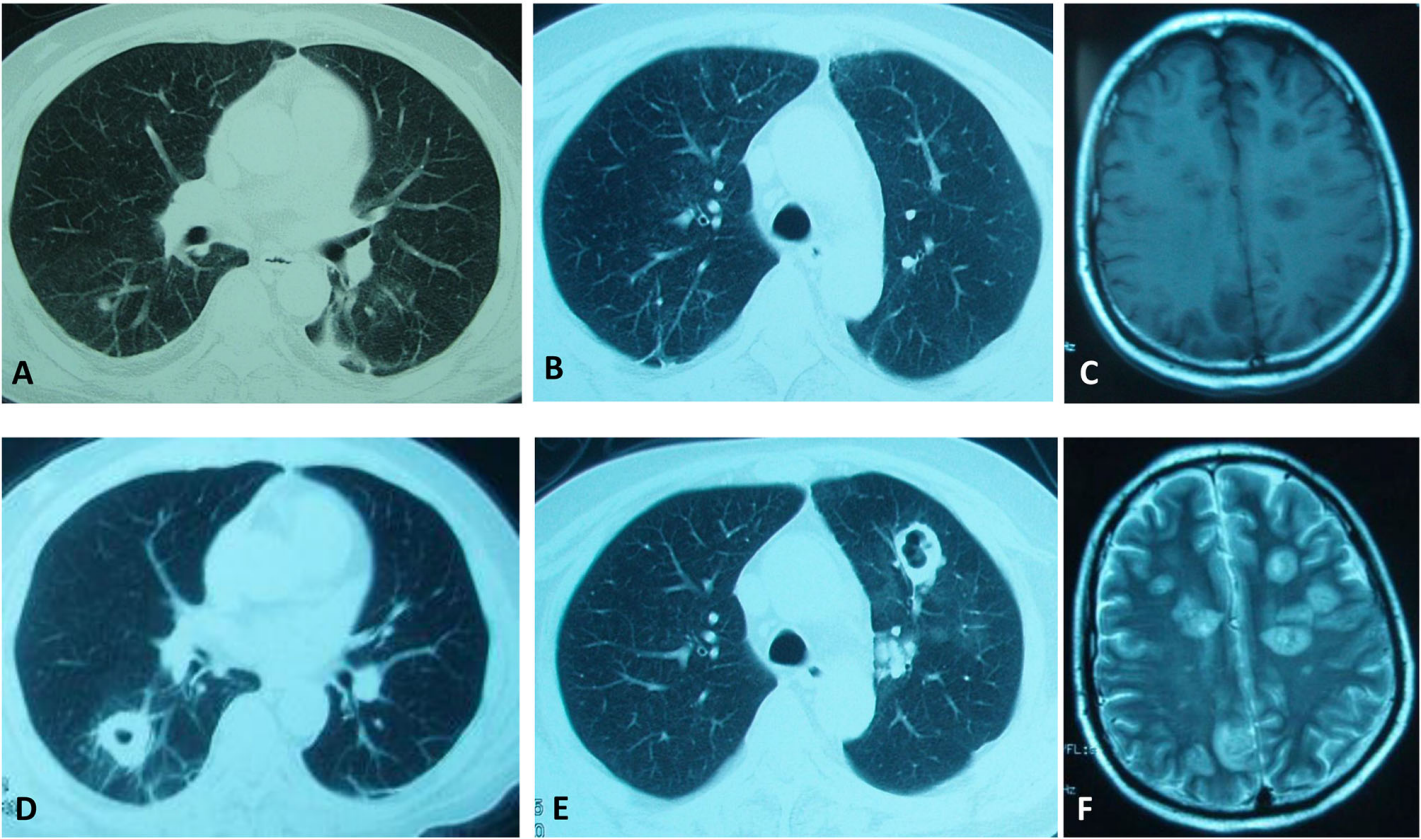
Clinical images of our patient. CT scanning of the chest showing continuous development to a large cavitary mass in both lung lobes. **(A, B)** were taken on the 6^th^ day. **(D, E)** were taken on the 15^th^ day. **(C, F)** MRI scanning showing multiple long T1/T2 signal lesions in the brain on the 24^th^ day.

A 41-year-old man developed fatigue for 2 months with progressive polydipsia and polyuria. A month ago, he went to the local hospital and had the examination revealed that his blood pressure was 150/100 mmHg and his fasting blood-glucose was 17 mmol/L. He was given insulin treatment afterwards. However, he could not uphold the regular treatment and his blood glucose could not be well controlled. He manifested fever of 38.4°C and cough with yellow phlegm following catching a cold 6 days ago. The patient gradually presented with mental disorders of mania and aggressive behavior for 4 days. He was urgently referred to our hospital. On admission, the man appeared weak and confused, thinning of the skin with pigmentation, bruising and edema of face and both lower extremities but no red-purple striae. He presented with mild moon face, no obvious buffalo hump. In laboratory examinations, serum glucose was 24.8 mmol/L, sodium 164 mmol/L, potassium 2.6 mmol/L, albumin 21g/L, creatine 102 μmol/L. Arterial blood gas analysis demonstrated of metabolic alkalosis. The X-ray of chest showed patch shadows of left middle lobe and right upper lobe. Insulin therapy, potassium supplements (oral potassium chloride, 9.0 g/d), spironolactone 60 mg/d, intravenous fluids, and empirical antibiotics moxifloxacin were applied. The patient’s consciousness, serum glucose, and sodium returned to normal 2 days later. He got a normal body temperature and less cough with phlegm 5 days later. On the 6^th^ day, chest CT showed patchy infiltration and small nodules of bilateral lung lobes (**Figures 1A, B**).

Examinations for CS were performed a week after his admission when the patient’s condition was improved. His serum ACTH was 171 pg/ml (normal range <46 pg/ml), 24-h urinary free cortisol (24hrUFC) was 3,522 μg (normal range 12.3–103.5 μg/24 h), repeated 24hrUFC was 2746 μg. The baseline serum cortisol was 51.21 μg/dl (normal range 4.0–22.3 μg/dl), after overnight 1 mg dexamethasone suppression test (DST) and high-dose DST were 58.76 and 62.74 μg/dl respectively. CS was diagnosed according to the clinical practice guideline of the diagnosis of CS (9). MRI scanning of pituitary gland showed no abnormal signal. In consideration of diabetes and hypertension in young age, mild physical appearance of CS, repeated high levels of 24hrUFC, and no suppression in 1 mg DST, the diagnosis of CS was established. Furthermore, because of rapid onset and severe conditions of the patient with the extreme cortisol excess, markedly elevated ACTH level, no suppression in high-dose overnight DST, no space-occupying lesion in pituitary gland, ectopic ACTH syndrome (EAS) was suspected. But CT scanning of chest and abdomen and ^99m^Tc-octreotide scintigraphy gave no clue for the ectopic location of ACTH-secreting tumor.

Chest CT on the 15^th^ day demonstrated multiple enlarged nodules, partial cavitary lesions (**Figures 1D, E**). Lung cancer was suspected but no tumor cell was founded in lung tissues from biopsy. GM test was positive. Modified acid-fast stains of sputum and lung tissues from percutaneous lung needle biopsy showed filamentous branching organisms. Sputum culture after 72 h grew *Aspergillus fumigates* and *Nocardia cyriacigeorgica*. Lung tissue culture after 48 h grew *Nocardia cyriacigeorgica*. Although trimethoprim-sulfamethoxazole (TMP-SMZ), ceftriaxone combined amphotericin B were used, the patient’s situation deteriorated with head MRI scanning displaying multiple long T1/T2 signal lesions suggesting multiple brain abscesses on the 24^th^ day (**Figures 1C, F**). We suspected the patient had the brain Nocardia or Aspergillus infections. Unfortunately, he refused further medication and died after auto discharge.

### Literature Review

Seventeen CS with nocardiosis patients from 15 published reports (8, 10–23) were reviewed. Together with our case, 18 CS patients (11 male, 7 female) were identified. Patients were HIV negative and had no history of organ transplantation, no use of immunosuppression therapy. The clinical characteristics were summarized in **Table 1**. The average age of patients was 41.9 ± 5.0 years. Eight patients (44.4%) had hypertension and 14 patients (77.8%) had diabetes mellitus. The causes of CS were all ACTH-dependent. Two patients (11.1%) were diagnosed as CD while nine patients (50.0%) diagnosed as EAS and seven patients (38.9%) with suspected as EAS of unknown origin including our patient. The ectopic ATCH originated from bronchial carcinoid (3/18), pancreatic neuroendocrine tumor (2/18), small cell lung carcinoma (1/18), paraganglioma (1/18), neuroblastoma (1/18), and small cell carcinoma in rib (1/18). The average 24hrUFC was 7,587.1 ± 2,772.0 μg/d. The average serum total cortisol and ACTH (8 AM) was 80.2 ± 18.7 μg/dl and 441.8 ± 131.8 pg/ml, respectively. Serum cortisol levels of 11 EAS and 2 CD were above 43.1 μg/dl. The 24hrUFC levels of 11 EAS were above 2,000 μg/d. Totally, serum cortisol or 24hrUFC levels were above these levels in 83.3% (15/18) patients.

**Table 1:** Clinical characteristics, diagnosis, treatments, and outcomes of 18 patients of Cushing’s syndrome with nocardiosis.

Authors	Country	Age (year)	Gender	HTN	DM	24hr UFC (μg)	ACTH (pg/mL)	Serum total cortisol (μg/dl)	Cause of Cushing’s syndrome	Infection sites	Chest imaging	Co-infection	Antibiotics	Treatment of Cushing’s syndrome	Outcome
Petersen DP, 1981 (10)	U.S.	72	F	No	No	NA	elevated	elevated	EAS origin from pulmonary carcinoid tumor	lung and skin	nodules	none	TMP-SMZ	Mitotane	mortality
Natale RB, 1981 (11)	U.S.	24	M	No	Yes	1,1820	902	110	EAS origin from bronchial carcinoid	lung	infiltrated and cavitary lesion	*Pneumocystis carinii*	TMP-SMZ	metapyrone and bilateral adrenalectomy	mortality
Higgins TL, 1982 (12)	U.S.	47	M	No	Yes	882	1,128	44	EAS origin from pancreatic neuroendocrine tumor	lung	nodules	*E. coli* and *Pseudomonas*	intravenous sulfadiazine and oral cycloserine	metyrapone, aminoglutethimide,5-fluorouracil, streptozocin, and Cytoxan	mortality
Findlay JC, 1992 (13)	U.S.	71	F	Yes	Yes	NA	NA	47.8	Cushing’s disease	lung and brain	cavitary lesion	none	sulfadiazine	aminoglutethimide and metyrapone	mortality
Boscaro M, 1994 (14)	Italy	27	M	No	No	980	48.5	27	occult EAS	lung, brain, and abdomen	infiltration	none	TMP-SMZ	metyrapone + aminoglutethimide followed by bilateral adrenalectomy	survival
Huang TP, 1994 (15)	China	25	M	No	Yes	8,454	725	62	EAS origin from rib small cell carcinoma	lung	nodules and cavitary lesion	none	NA	ketoconazole	mortality
Beinart GA, 2003 (16)	U.S.	68	M	Yes	Yes	4,322	519	82	EAS origin from metastatic small cell lung carcinoma	lung	consolidation and cavitary lesion	*Aspergillus*, *Clostridium difficile* colitis, enterococcal bacteremia	TMP-SMZ	carboplatin, etoposide, ketoconazole	mortality
Chrysanthidis T, 2010 (17)	Greece	52	F	No	Yes	>1812	79	20.3	occult EAS	lung, brain, and skin	infiltration	none	meropenem, gentamicin, and minocycline	ketoconazole	mortality
Sutton BJ, 2011 (18)	U.S.	42	F	No	No	NA	152	NA	EAS origin from pulmonary carcinoid tumor	lung	nodules	none	TMP-SMZ	RFA of the carcinoid tumor	survival
Chowdry RP, 2012 (19)	U.S.	48	F	No	Yes	16,340	296	106.2	EAS origin from pancreatic neuroendocrine cancer	lung and blood	nodules and pleural effusion	*E. coli* and *Pneumocystis jirovechi*	TMP-SMZ	ketoconazole	mortality
Momah N, 2012 (20)	U.S.	42	M	Yes	Yes	21,469	1,013	130	occult EAS	lung and brain	cavitary lesion	methicillin-sensitive *Staphylococcus aureus*, Pneumocystosis and brain aspergillosis	TMP-SMZ	ketoconazole, octreotide, and radical thymectomy and mediastinectomy	mortality
Rizwan A, 2014 (21)	Pakistan	53	M	Yes	Yes	2,000	68.5	20	occult EAS	lung	cavitary lesion	none	TMP-SMZ	bilateral adrenalectomy	survival
Rizwan A, 2014 (21)	Pakistan	54	M	Yes	Yes	27,216	159	134	occult EAS with multiple metastasis	lung	cavitary lesion	none	TMP-SMZ	none	mortality
Rizwan A, 2014 (21)	Pakistan	38	M	Yes	Yes	9,088	255	192	occult EAS	lung	consolidation and pleural effusion	none	TMP-SMZ	ketoconazole	survival
Xu L, 2016 (8)	China	35	M	Yes	Yes	3,118.08	372	>50	EAS origin from mediastinal paraganglioma	lung	nodules and cavitary lesion	none	TMP-SMZ	resection of the mediastinal tumor	survival
Kobayashi K, 2018 (22)	Japan	52	F	No	No	NA	469	59.6	EAS origin from olfactory neuroblastoma	lung	nodules	none	TMP-SMZ	metyrapone and mitotane	survival
Mylonas CC, 2019 (23)	Greece	40	F	Yes	Yes	NA	126.9	61.5	Cushing’s disease	lung	nodules and cavitary lesion	none	TMP-SMZ	transsphenoidal pituitary surgery	survival
Our case, 2020	China	41	M	No	Yes	3,522	171	51.2	occult EAS	lung and brain	cavitary lesion	*Aspergillus*	TMP-SMZ, ceftriaxone and amphotericin B	none	mortality

The pulmonary nocardiosis related symptoms varied including fever, cough, expectoration, dyspnea, chest pain, and hemoptysis. Seven patients were confused and six patients progressed to respiratory failure that required intubation and mechanical ventilation. Three patients had only chest imaging changes with no fever or any pulmonary symptoms. The pulmonary radiologic findings included cavitary lesions (10/18), nodules (8/18), infiltration (3/18), consolidation (2/18), and pleural effusion (2/18) (**Table 1**). Multiple pulmonary radiologic findings manifested in one patient. The diagnosis of nocardiosis was established by modified acid-fast and/or methenamine silver stain and culture from sputum, bronchoalveolar lavage fluid (BALF), or biopsy tissues of lung, skin, and brain lesions. Pulmonary nocardiosis was diagnosed in all patients. Other infection sites of Nocardia were brain (5/18), skin (2/18), blood (1/18), and paravertebral site (1/18). Co-infections were found in 33.3% (6/18) patients. Co-infected microorganisms included *Pneumocystis jirovechi* (3/18), *Aspergillus* (3/18), *Escherichia coli* (2/18), *Clostridium difficile* (1/18), *Enterococcus* (1/18), *Pseudomonas* (1/18), and *Staphylococcus aureus* (1/18) (**Table 1**). Diagnosis time of Nocardia was variant. The shortest time for identification of Nocardia was 3 days after symptoms onset. In some patients, the Nocardia identification time lasted for several weeks, even after the patients’ death.

Fourteen patients were treated with TMP-SMZ for nocardiosis. Due to the resistance of TMP-SMZ, one patient was treated with meropenem and gentamicin. The duration of antibiotic therapy lasted from 3 days to 1 year. The treatment of CS included surgery and medical therapy. Transsphenoidal pituitary surgery was performed in one patient, resection or radiofrequency ablation (RFA) of EAS tumor in three patients, bilateral adrenalectomy in three patients. Drugs that reduced cortisol levels, including ketoconazole, metapyrone, mitotane, and cytotoxic drugs, were used in 12 patients. In terms of prognosis, 11/18 (61.1%) patients died. Eight patients died of infections and three patients died of progression of malignancy. The average ACTH, cortisol, and 24hr UFC level of mortality and survival were 554.7 ± 401.0 and 213.1 ± 159.0 pg/ml (*P* = 0.04), 78.8 ± 39.5 and 72.0 ± 69.6 μg/dl, 9,369.3 ± 9,560.8 μg, and 3,796.5 ± 3,634.1 μg, respectively. The patients that had co-infections had higher ACTH level (671.5 ± 398.2 *vs* 245.5 ± 217.1 pg/ml, *P* = 0.047). Patients with lower level of ACTH, no co-infection, underwent CS surgery and received antibiotic therapy for more than 6 months had more possibilities to survive (**Table 2**).

**Table 2:** Comparison of clinical characteristics between distinct outcomes of patients of Cushing’s syndrome with nocardiosis.

		Survival	Mortality	P value
n		7	11	
Age (year)		41.0 ± 9.2	49.5 ± 16.5	0.24
Gender	Female	3	4	0.78
	Male	4	7	
24hrUFC (μg)		3,796.5 ± 3,634.1 (n = 4)	9,369.3 ± 9,560.8 (n = 6)	0.31
F (μg/dl)		72.0 ± 69.6 (n = 5)	78.8 ± 39.5 (n = 10)	0.81
ACTH (pg/ml)		213.1 ± 159.0 (n = 7)	554.7 ± 401.0 (n = 9)	0.04
Cause of Cushing’s syndrome	CD	1	1	1.00
	EAS	6	10	
DM	Yes	4	10	0.25
	No	3	1	
Extrapulmonary	Yes	1	6	0.09
nocardiosis	No	6	5	
Co-infections	Yes	0	6	0.02
	No	7	5	
Surgery of CS	Yes	5	2	0.02
	No	2	9	
Treatment duration	≥6 months	6	2	0.002
of antibiotics	<6 months	0	8	

## Discussion

Although nocardiosis in EAS patients has been reported, our review presented 18 nocardiosis with CS patients (16 EAS and 2 CD) and emphasized the possibility of Nocardia infection in other forms of CS. In addition, according to the clinical characteristics, treatments, and prognosis of these 18 patients, we put forward the risk factors for mortality in CS patients with nocardiosis.

Opportunistic infections in endogenous CS were predominantly observed in patients with severe cortisol excess (24). Previous reports (16, 25) had shown that high levels of endogenous glucocorticoids above the cut-off levels of serum cortisol, 43.1 μg/dl and 24hrUFC, 2,000 μg/d, were reliable indicators for severe infections in EAS patients. Fifteen of 18 (83.3%) patients including 13 EAS and 2 CD patients in our series were detected of high levels of serum cortisol or 24hrUFC exceeded these cut-off values. In addition, our review showed that CS patients with higher level of ACTH had more risks for co-infections and mortality. It was suggested that we should give more concern to avoiding infections in CS patients with extremely high ACTH concentrations. We did not find the difference in serum cortisol or 24hrUFC between patients of survival and mortality maybe because of the relatively small sample size. Hypercortisolism impaired cellular and humoral immunity. CS patients show significant lymphopenia, especially the reduction in the CD4+ subset, the reduction in the CD4/CD8 ratio are predictors for opportunistic infections (26). However, there was no record of lymphocytes subsets analysis in our review.

Pulmonary infection was the most common manifestation in nocardiosis. The clinical characteristics and symptoms of pulmonary nocardiosis were non-specific. Some patients had no pulmonary symptoms while some patients experienced respiratory failure rapidly. The radiologic findings were variable. Nodules, masses, cavitations, infiltration, consolidation, and pleural effusion could be radiographic presentations of pulmonary nocardiosis. Xu L. et al. (8) proposed cavity lesions, consolidation/infiltration, and nodule/mass were the major findings for EAS patients. The most common findings were cavitary lesion and nodules in our review. It was noted that these radiologic findings could also be the presentation of fungal, mycobacterial infections, and malignancies including both primary and metastatic lung cancers. Lung nodules were suspected to be tumors of EAS in five patients (27.7%) (19, 21–23) in our series including our patient. Biopsies of suspicious lung nodules were performed. Histological and cytologic examination of the biopsy showed no evidence of malignancy but inflammation. Nocardia infection was confirmed by the biopsy. Therefore, rapid changes of chest imagings indicated an infective etiology rather than malignancy and Nocardia infection should be carefully cautioned (19).

Aggressive diagnostic approaches were warranted in individuals suspected of infections. Broncho-alveolar lavage (BAL), brushing by bronchoscopy, or percutaneous lung fine-needle aspiration from the cavitated nodule might be the drawing location for cytology examinations and culture to establish the diagnosis of pulmonary nocardiosis. We should pay adequate attention in order to make early etiological diagnosis.

In our CS review, 11 of 18 patients (61.1%) died. The mortality rate was similar with that reported in EAS patients of 66.7% (8). It is seemed that the mortality rate of nocardiosis in CS patients is higher than that in other immunocompromised patients of 34.5–40% (3, 4). Mortality appeared to be correlated with multiple sites of infections and was reported as high as 100% in patients with disseminated diseases (4, 27). We did not find the extrapulmonary nocardiosis had impacts on mortality. The reason might be the small sample of our case series. Co-infections with other microorganisms have been found to attribute to mortality in nocardiosis (28). Our patients and the other two patients (16, 20) with nocardia and aspergillus co-infections had bad outcomes. Moreover, 33.3% (6/18) patients had co-infections in our series. All of them died afterwards. CS patients with marked high levels of ACTH are prone to have co-infections. Clinicians need to be mindful of opportunistic co-infections in patients with CS.

Reducing the cortisol level was essential for the treatment of CS with nocardiosis (29, 30). Resection of primary tumor that induced over-secretion of ACTH was an efficient and rapid strategy. However, EAS can be a diagnostic challenge with the hormonal source difficult to find. Seven patients (38.9%) had occult EAS in our series. ^68^Ga-conjugated somatostatin receptor targeting peptide positron emission tomography (^68^Ga-SSTR-PET/CT) contributes to localization of primary tumor of EAS (31). If the primary tumor couldn’t be found, bilateral adrenalectomy might be of value (32). Anticortisolic drugs also provided decrease of hypercorticism (33).Patients who underwent CS surgery had better prognosis than those treated by medicines only. Moreover, it was worth mentioning that patients with nocardiosis generally needed 6 to 12 months of antibiotic therapy, depending on their immunological status and the organs infected (34). The survived patients received antibiotic drugs for more than 6 months in our review.

There are some limitations in this study. Firstly, EAS was suspected without definite localization of primary tumor produced excess hormone in our patient. Secondly, this is a retrospective study. In addition, the sample size is relatively small as Nocardia infection in CS is incredibly rare reported. Future research is required to improve the prognosis of CS with nocardiosis.

In conclusion, Nocardia infection should be cautioned when a patient with CS presents abnormal chest radiographs. The mortality risk factors of CS with nocardiosis are high level of ACTH and co-infections. We should endeavor to make early etiological diagnosis. Long-term application of sensitive antibiotics and aggressive treatments of CS are beneficial for prognosis.

## Data Availability Statement

The original contributions presented in the study are included in the article. Further inquiries can be directed to the corresponding author.

## Ethics Statement

The studies involving human participants were reviewed and approved by Peking Union Medical College Hospital. The patients/participants provided their written informed consent to participate in this study. Written informed consent was obtained from the individual(s) for the publication of any potentially identifiable images or data included in this article.

## Author Contributions

DZ and YJ designed the study, and DZ and HF participated in data collection. DZ performed the systematic review and drafted the manuscript. YJ edited and reviewed the manuscript. LL, ZL, WX, and XX partially conceived the research idea. All authors contributed to the article and approved the submitted version.

## Funding

This study was supported by grant from the Non-profit Central Research Institute Fund of Chinese Academy of Medical Sciences (2018PT32001) and grant from Peking Union Medical College Hospital (ZC201904197).

## Conflict of Interest

The authors declare that the research was conducted in the absence of any commercial or financial relationships that could be construed as a potential conflict of interest.
